# Influence of Screw Length and Bone Thickness on the Stability of Temporary Implants

**DOI:** 10.3390/ma8095322

**Published:** 2015-09-23

**Authors:** Daniel Jogaib Fernandes, Carlos Nelson Elias, Antônio Carlos de Oliveira Ruellas

**Affiliations:** 1 Laboratory of Biomaterials, Instituto Militar de Engenharia, Rio de Janeiro, RJ 22290-270, Brazil; E-Mail: elias@ime.eb.br; 2 School of Dentistry, Universidade Federal do Rio de Janeiro, Rio de Janeiro, RJ 21941-902, Brazil; E-Mail: antonioruellas@yahoo.com.br

**Keywords:** stability, cortical thickness, temporary screws, torque, bone

## Abstract

The purpose of this work was to study the influence of screw length and bone thickness on the stability of temporary implants. A total of 96 self-drilling temporary screws with two different lengths were inserted into polyurethane blocks (n = 66), bovine femurs (n = 18) and rabbit tibia (n = 12) with different cortical thicknesses (1 to 8 mm). Screws insertion in polyurethane blocks was assisted by a universal testing machine, torque peaks were collected by a digital torquemeter and bone thickness was monitored by micro-CT. The results showed that the insertion torque was significantly increased with the thickness of cortical bone from polyurethane (p < 0.0001), bovine (p = 0.0035) and rabbit (p < 0.05) sources. Cancellous bone improved significantly the mechanical implant stability. Insertion torque and insertion strength was successfully moduled by equations, based on the cortical/cancellous bone behavior. Based on the results, insertion torque and bone strength can be estimate in order to prevent failure of the cortical layer during temporary screw placement. The stability provided by a cortical thickness of 2 or 1 mm coupled to cancellous bone was deemed sufficient for temporary implants stability.

## 1. Introduction

Different miniscrew designs are available for diverse biomedical applications, including orthopaedics and neurosurgery in medicine and orthodontics and maxillofacial surgery in dentistry [1,2]. Regardless of the application, the screw performance is strongly correlated with bone quality [2]. An acceptable bone quality classification considers both cortical and trabecular bone and is based on the ratio of cortical to cancellous bone in the host region [3]. Several bone quality classification systems have been proposed that are based largely on anatomical and histological data. Lekholm and Zarb proposed four groups (types) of bone: bone D1, mainly homogeneous compact cortical bone; D2, a thick layer of compact cortical bone surrounding a dense trabecular bone core; D3, a thin layer of cortical bone bordering a lower dense trabecular bone core and, D4, a thin layer of cortical bone environing a reduced density trabecular core [4].

Different works have analyzed the correlation between stability [5] and the most appropriate jaw region for miniscrew placement for orthodontic treatment [6]. No difference in success rate was credited to reduction in miniscrew diameter from 2.3 to 1.5 mm; however, a significant influence in stability was attributed to miniscrew length when associated with bones with adequate density for immediate attachment purposes [7]. Disregarding bicortical insertion, which is mainly available in craniofacial regions, monocortical placement of temporary screws is primarily stabilized by the amount of dense cortical bone present in the attachment area [5,6,8]. So, it seems logical to consider the possibility of decreasing the screw length in order to facilitate the surgical procedure. Other advantages involve reduction of anatomic bone limitation during surgical access, less discomfort and reduced risk of implant fracture or bone damage during screw attachment [7]. 

The purpose of this work was to evaluate the influence of cortical and cancellous bone thickness on the stability of temporary biomedical screws used in dentistry and with two different lengths. The bone response to stress distribution was monitored by micro-CT.

## 2. Materials and Methods

**Figure 1:**
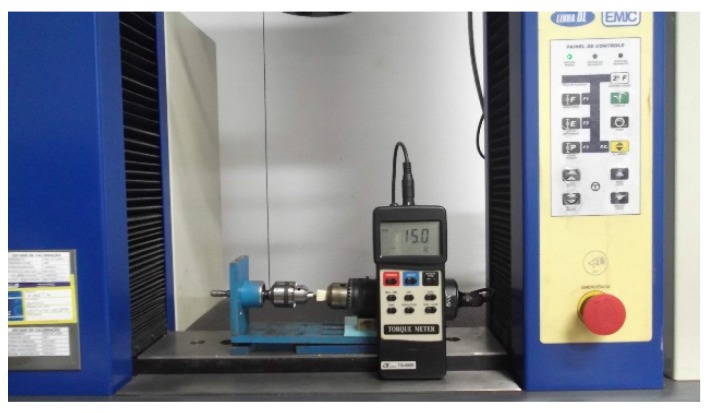
The set up used for insertion and remove screws torque test.

A total of 96 screws, made of Ti-6Al-4V alloy (ASTM Ti G5) with 1.5 mm in diameter and two different lengths (6 and 8 mm), supplied by Conexão Co (Conexão Sistema de Próteses, Arujá, SP, Brazil) were used. The screws sites were drilled with a 1 mm diameter pilot drill with the same depth of each screw length. The screws were inserted according to ASTM F117 (ASTM F117, Test Method for Driving Torque of Medical Bone Screws) and F543 (F543-13e1, Standard Specification and Test Methods for Metallic Medical Bone Screws). Screws were inserted by an Emic DL10000 universal testing machine (Emic Co – São José dos Pinhais, Brazil) with a 500 N load cell, 4 N compression, a displacement of 1 cm/min, and the system had an accuracy of 0.02 N·cm during insertion torque test. The insertion torque was confirmed by a digital torquemeter (Lutron TQ-8800, Taipei, Taiwan) with an accuracy of ±0.02 N·cm. Figure 1 shows the set up used for insertion and remove screws torque test.

In order to study the influence of screw length and bone thickness on the mechanical stability of the temporary implants, the screws were inserted into synthetic bone (polyurethane composite blocks) and natural bone (cadaver bovine femurs and New-Zealand rabbit tibia). The use of synthetic bone substrates arose from the fact that the insertion torque into natural bone may have a large dispersion due to bone heterogeneity. 

Rigid polyurethane (Nacional Co, Jaú, SP, Brazil) with two different densities were used (ASTM F1839/2012) to emulate natural bone properties of cortical bone (0.64 g/cm^3^) and cancellous bone (0.32 g/cm^3^).

A total of 66 screws were inserted into different synthetic bone substrate, from where each group was composed by 6 screws. Screws with 6 mm and 8 mm in length were inserted in composite with three different cortical thickness (1, 2 and 3 mm), totaling 36 screws. Both screw lengths were completely inserted in dense polyurethane blocks with only cortical bone, summing 12 screws. In order to disclose cancellous bone role, 6 mm screws were attached to cortical blocks with three different thicknesses (1, 2 and 3 mm), totaling 18 screws. 

In natural bone sources, 30 screws with 6 mm in length were used in bovine (n = 18) and rabbit (n = 12) bones. Six screws were attached in three different cortical thicknesses (2.5, 3.5 and 4.5 mm) of bovine femur, while six screws were placed into each rabbit tibia and cortical thickness was evaluated by micro-CT. This study was approved by the Rio de Janeiro State University Committee regarding ethical and animal care.

**Figure 2:**
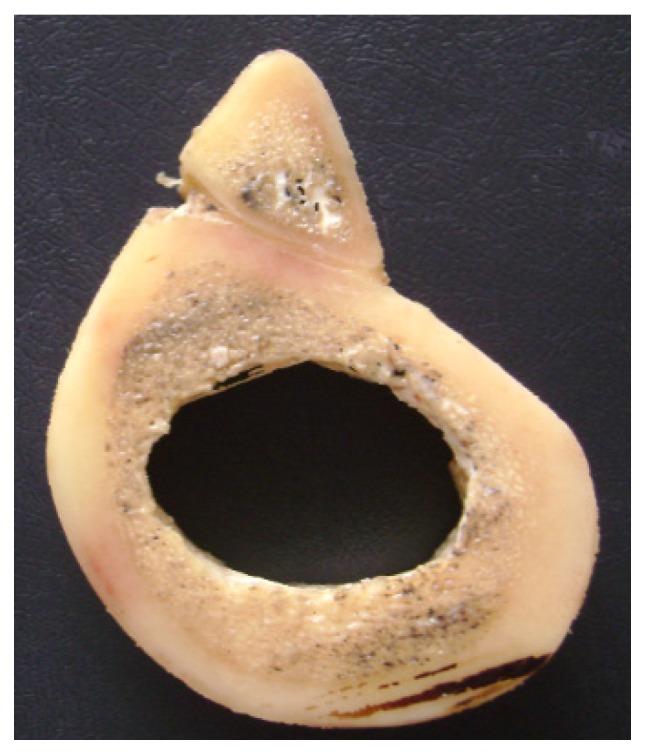
Transversal section of a bovine femur. Cortical and cancellous bone can be easily distinguished.

Two fresh bovine femurs of cattle about 30 months old were obtained from a butcher. The tibias were cleaned and sliced in the transverse direction (Figure 2). Samples discs were cut and cortical thickness selections were performed in order to reduce variations in density, mineral content and cortical thickness. The samples were stored in a refrigerator (T = 4 °C) and immersed in a solution of 70% ethanol in water for 48 h.

Two 6-month-old female New Zealand white rabbits (1.8–2.0 kg) were used. Six screws were inserted into each rabbit tibia. All surgical procedures were conducted under systemic (0.2 mg/kg ketamine and 3 mg/kg xylazine IM) anesthesia. Hair on the medial surface of the upper inner tibia was clipped and an incision was made parallel to the longitudinal orientation of the tibia. Three screws were placed in each tibia. After a healing period of 1 week, all animals were euthanized and samples of bone were cut and scanned with a high-resolution microtomography system (micro-CT) in the multislice mode and the cortical thickness was measured.

## 3. Results

**Figure 3:**
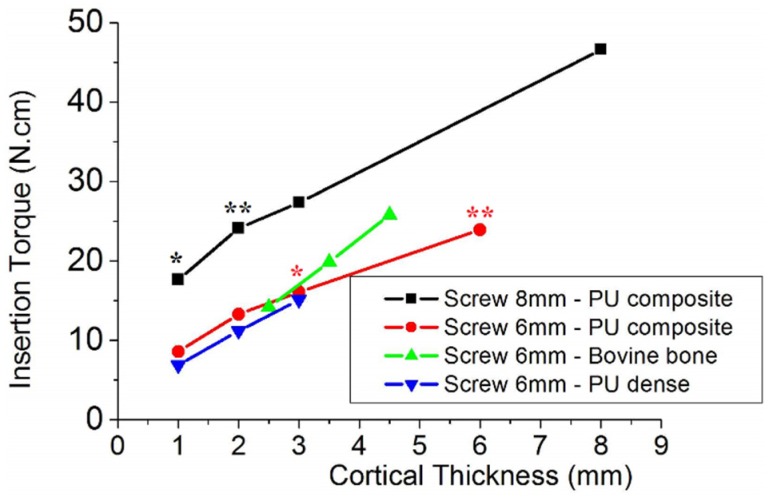
Insertion torque of screws into synthetic bone substrate and bovine bone with different thicknesses. The absence of a statistically significant difference between torques (p > 0.05) is indicated by (*) and (**).

Figure 3 shows that the insertion torque increases linearly with the thickness of the cortical bone for both screw lengths. This relation was observed for 6 and 8 mm screws inserted into polyurethane composite, which simulated cortical bone with cancellous bone; and dense polyurethane, which simulated just cortical bone. For polyurethane composites with the same cortical thickness, the insertion torque almost doubled when the screw length was increased from 6 to 8 mm Table 1). Comparison of insertion torque values between these groups (red and black lines) was performed by an analysis of variance and a Tukey’s post-hoc test at p = 0.05. Statistically differences (p < 0.0001) from ANOVA were confirmed by Tukey’s test among all groups (p < 0.05) (Table 1). The absence of a statistically significant difference between torques (p > 0.05) was indicated by (*) and (**) in Figure 3.

**Table 1:** Mean insertion torque (N·cm) and standard deviation (SD) of 6 mm mini-implant screw length inserted into polyurethane composite with different cortical thickness.

Cortical	Insertion Torque
1 mm	6.9 (0.3)
2 mm	11.2 (0.9)
3 mm	15.1 (0.6)
Cancellous	1.8 (0.4)

ANOVA (p < 0.0001). Tukey’s multiple comparison test among all rows (p < 0.05).

Table 2 shows the insertion torque into bovine tibia. Insertion torque increases as the cortical bone thickness increases. The statistical analysis shows that the insertion torques are different among these groups (p = 0.0035). 

**Table 2:** Mean values torque (N·cm), standard deviations (SD) and cortical thickness (mm) influence of 6 mm length screw placed into bovine femur.

Cortical	Torque	Tukey’s Test
2.5 mm	14.18 (2.38)	A
3.5 mm	19.87 (5.64)	A,B
4.5 mm	25.80 (5.85)	B

The data shown in Figure 3 may be represented by Equations (1)–(3). Where *x* is the cortical bone thickness, *T*_6PU_ and *T*_8PU_ represent the insertion torque of 6 and 8 mm screws into polyurethane substrate, respectively and *T*_6Bov_ is the insertion torque of 6 mm screws into bovine bone. Considering that the cortical bone thickness is equal to zero, which means that the substrate has only cancellous bone, the insertion torque of 6 and 8 mm screws into polyurethane is 6.61 and 14.97 N·cm, respectively. These results show that screw length influences the primary stability of temporary screws inserted into cancellous bone.

*T*_6PU_ = 2.96 *x* + 6.61 (1)

*T*_8PU_ = 3.99 *x* + 14.97 (2)

*T*_6Bov_ = 5.72 *x* + 0.05 (3)

Based in the results showed in Figure 3, it was also observed the influence of cortical and cancellous bone substrate in insertion torque. 

Table 3 shows the cortical thickness of rabbits and the insertion torque. Although the cortical thickness of the second rabbit was twice of first rabbit, the insertion torque increased only 43.6%. Significantly interactions between the insertion torque and the cortical thickness of the rabbit tibias were showed by the Bonferroni’s test with p < 0.05, for both samples.

**Table 3:** Mean values (standard deviations) of cortical bone thickness (mm) of rabbit tibia measured by micor-CT scans and insertion torque (N·cm) of 6 mm screw length. Correlation between same rows were performed by an analysis of variance with Bonferroni’s test at p = 0.05.

Cortical thickness	Torque	Bonferroni’s Test
1.50 (0.05)	10.20 (1.06)	p < 0.05
3.20 (0.04)	14.65 (2.14)	p < 0.05

## 4. Discussion

The main purpose of this paper was to evaluate the influence of bone quality on the stability of temporary screw implants with different lengths. The motivation was to achieve an optimal balance between screw length and mechanical stability, in order to reduce, as much as possible, the screw length without compromising the stability of the implants. Marquezan *et al.*, performed a systematic review of miniscrew implants and reported a positive relation between the primary stability of screws and cortical thickness. They also stated that none studies performed in animals was included in their systematic review because it was toilsome to group different bone tissue from different animal sources [9].

Some works studied the primary stability of screws measuring the insertion torque into synthetic and natural bone [8,10,11,12,13,14]. In the present paper, different bone tissues were grouped according to measurements of cortical and cancellous thickness. Results from micro-CT scans for *in vivo* samples and direct measurements from cadaveric and synthetic bone samples allowed insertion torque comparisons. 

Chang *et al.* and Schatzle *et al*. expressed their concern upon the use of insertion torque as a unique indicative of screw stability. A high insertion torque can induce cracks and even necrosis in the bone [10,15]. In the present work, we used this method to measure the insertion torque into synthetic bone (polyurethane with different densities) and cadaveric bone (from bovine tibias) and *in vivo* animal (tibia of rabbits). The purpose was to analyze the possibility of screw length reduction and allow a surgery process easy for surgeons and safer for patients due to small screw sizes [12,16]. Although insertion torque is considered a well-established technique to quantify the mechanical stability, the authors of the present work proposal a new method for quantify the implant stability, which is named insertion strength.

Different works have considered the influence of screw design, diameter and length on its efficiency as temporary devices [6,7,10,12,13,17]. Considering that the implant screw design varied among manufacturers, we decided to analyze the influence of screw length on its stability employing the same screw design.

**Figure 4:**
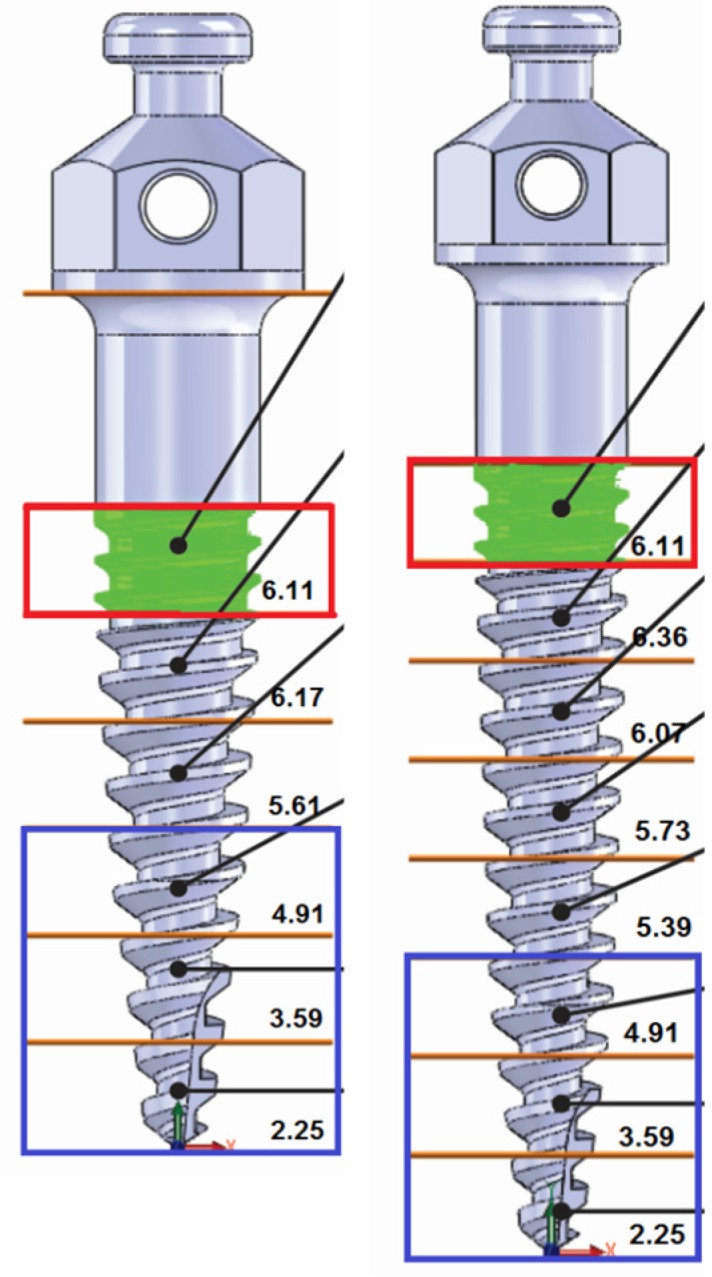
Temporary screws with 6 (**left**) and 8 mm (**right**) length. Slices were performed at each 1 mm from the tip. Same colors indicate matching in surface area from different screws. The red rectangle shows the screw length that is inserted into cortical bone.

**Figure 5:**
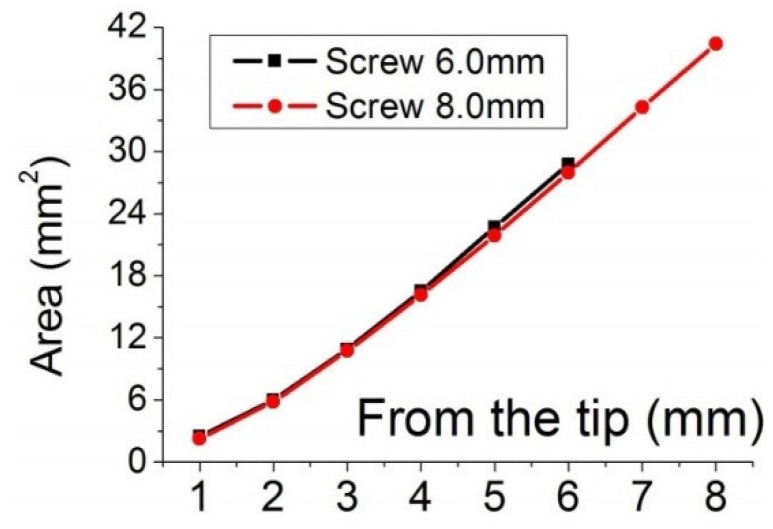
Dependence of the surface area on length.

**Figure 6:**
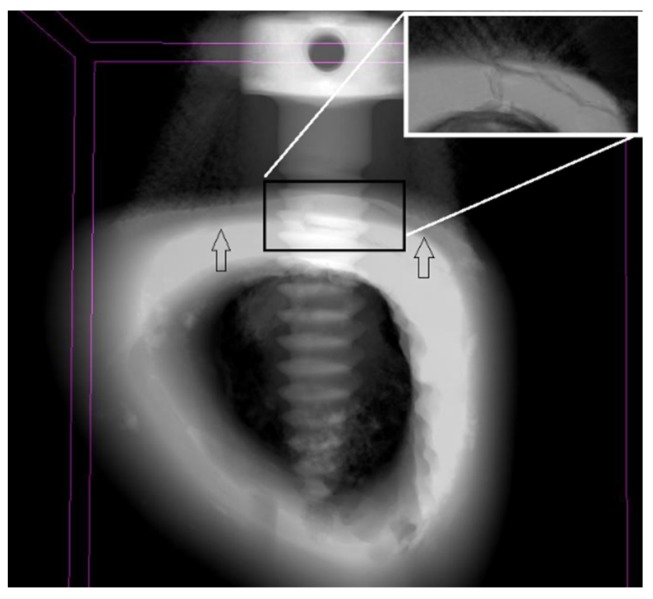
Micro-CT scan of the temporary screws attached to rabbit’s tibia after 1 week. Cortical and cancellous bone limits are identified by arrows. Detail of fractured bone due high stress induced during mini-implant insertion at increasing magnification.

A CAD-CAM software was used to calculate of the surface area of the screws. The screws were sliced at each 1.0 mm from the tip and the surface area was calculated (Figure 4 and Figure 5). Figure 4 shows the screw surface of each 1 mm length. In Figure 4, the red box is the screw part that was in contact with high polyurethane density (cortical bone). The difference between the total surface areas of two screws was 11.79 mm^2^, which could imply more friction with the bone and may explain the higher insertion torque measured for 8 mm screws (Figure 6). Figure 5 shows a comparison of surface areas.

Figure 3 shows a statistical similarity of insertion torque among screws with different lengths attached to different composite polyurethane foams. The symbol * indicates no statistical difference in insertion torque between 8 mm screws inserted into 1 mm of cortical density and 6 mm screws inserted into 3 mm of cortical density, although the total area of 6 mm screws (11.78 mm^2^) is lower than that of 8 mm screws (23.57 mm^2^).

No statistical difference (symbol ** in Figure 3) was observed between 8 mm screws inserted into 2 mm cortical (and 6 mm cancellous) polyurethane and a 6 mm screw inserted into a substrate of dense foam (cortical density polyurethane). Assuming that cortical density polyurethane is two times more resistant to insertion torque than cancellous density foam, a 6 mm cancellous bone layer may be replaced by a 3 mm cortical bone without significant changes in insertion torque. Thus, an 8 mm screw inserted into 2 mm cortical bone will have the same stability as a 6 mm screw inserted into a 5 mm cancellous bone substrate and 1 mm cortical bone. Therefore, 1 mm of cortical bone thickness would be not sufficient to provide relevant stability. This statement is consistent with the data presented in Table 2, from where changes in stability were significant (p = 0.0035) just above 2 mm increase in cortical thickness (from 2.5 to 4.5 mm). 

Figure 3 shows the influence of cortical (or dense polyurethane substrate) thickness in the insertion torque of screws with different lengths. As expected, the insertion torque of 8 mm screws was greater than that of 6 mm screws due to differences in surface area. Besides that, it was interesting to consider in Figure 3 that the two lines had almost the same slope, which represents the role of bone density on insertion torque, since both composite foams had the same ratio of cortical/cancellous density polyurethane foams. Based on this finding, the larger slope of the line for bovine bone may be attributed to the greater density of bovine bone than that of polyurethane foams. Another important finding that arose from Figure 3 was the role of cancellous bone on the insertion torque. Comparing lines related to 6 mm screws attached in composite and dense polyurethane, the slope was almost the same, which means that cancellous bone compensated the greater cortical density participation in insertion torque. The evaluation of cancellous bone contribution to stability was reviewed by Marquezam *et al*. [18]. They also stated that although a positive correlation was seen in different works between bone density and stability, there was a lack of evidence of this statement upon temporary screws, which take our work one step forward in the attempt to reveal the cortical/cancellous bone participation in temporary screws stability [9,18].

Reiterating the inclusion of one more variable instead of just insertion torque as a stability predictor, the authors of the present work suggest a new parameter named insertion strength. Insertion strength is commonly calculated by pullout tests [19]. The screw pull out test results are a function of bone density and screw design. Instead of obtaining these values from a pull out test, the authors of the present work propose to measure the shear forces and insertion strength during implant placement. The shear force is the friction force between the screw threads and bone.

Considering both effects of the cortical and cancellous bone thickness in the insertion strength, the shear stress is given by Equation (4).

(4) $$𝜏=\frac{T}{\text{π}d^{2}\left({\text{μ}}_{1}h_{1}+\;{\text{μ}}_{2}h_{2}\right)}$$

where 𝜏 is the insertion strength in MPa, *T* is the insertion torque in N·cm, *d* is the major diameter of the screw in mm, *h* is the length of the part of the screw inserted into bone tissue in mm, μ is the friction coefficient and the subscripts 1 and 2 mean cortical bone and cancellous bone, respectively.

Reckoning the constant values presented above and Equation 4, we find the insertion strength 𝜏 = 1.01 MPa. Insertion strength provides an indication of the stress on bone tissue, mainly cortical shell. Knowing the insertion strength, it is possibly to avoid bone fracture during screw placement. Despite the values of insertion torque measured in Table 1 were in accordance with the baseline disclosed in literature for adequate success rate of biomedical temporary screws, the importance of these calculations lies in the ability of the surgeon to select the correct diameter of screws in order to avoid cortical fracture during attachment [20,21]. From our calculations, for 1.5 mm screw diameter, the stress that bone can stand is close to the value transmitted during screw placement. The correlation coefficient between insertion strength and torque was just 1.01. This means that for the conditions of our experiment, cortical density strength is slightly higher than insertion torque brought forth during screw placement. Although these calculations were performed in synthetic foam, it is consistent with our results from *in vivo* tibia of rabbits, where an example of cortical failure was identified on the outer region of the tibia (Figure 6). In order to prevent cortical fracture, surgeons ought to choose an adequate design, length and diameter pf the screw according to bone quality, insertion technique (with or without use of 1.0 mm drill before insertion) and angle of penetration [13,17,19,22,23,24,25,26,27].

Zioupos *et al*., pointed out that there are differences in cancellous and cortical mechanical resistance and in the maintenance of its integrity [28]. Cancellous bone has lower number of trabeculae, smaller susceptibility to strain and damage than cortical bone. Combining this statement of bone stiffness and density with the anisotropic comportment of trabecular bone when loaded with multiaxial loads *in vivo* [29], our experimental data of insertion torque should be considered might sensitive to bone tissue density. Thereby, for higher densities bone, a greater axial load is necessary to overpass the higher mechanical resistance imposed by the bone tissue surface. Based on insertion torque (Table 1), increasing only the cortical thickness, each screw millimeter length increases the insertion torque at 60%. Although the influence of cancellous density polyurethane foam was much less significant than cortical layer, the sum of both torque values was in agreement with insertion torque results of composite foams presented in Figure 3.

## 5. Conclusions

The influence of cortical thickness and the length of temporary screws on stability was evaluated. The main findings were the following:

(1) A direct relation exists between cortical thickness and insertion torque.
(2) The insertion torque of 8 mm screws is twice that of 6 mm screws.
(3) For clinical needs, a cortical thickness of 1 mm coupled with cancellous bone provided sufficient mechanical stability, while a cortical thickness above 2 mm was necessary when cancellous bone substrate was not considered.
(4) For cortical thickness around 2 mm, insertion strength and bone fracture values were close to insertion torque, which means that the amount of stress transmitted should be considered in order to prevent outer cortical fracture.
(5) Stability provided by temporary screws of different lengths might encourage manufacturers to reduce their length, since the stability provided by cortical with reduced thickness seemed sufficient for clinical needs.
